# Supplementary material for: Antihypertensive Drugs and the Risk of Cancer: A Nationwide Cohort Study
Source: J Clin Med. 2021 Feb 15;10(4):771. doi: 10.3390/jcm10040771 (PMC7918966; doi:10.3390/jcm10040771)
Supplement: Supplementary file 1 [file jcm-10-00771-s001.pdf]

Subgroup	Overall Cancer Incidence		Hazard Ratio* (95% CI)	P value for interaction
	ARB non-user	ARB user		
	no./total (%)			
**Age**				
< 60 yr	521 (42.7)	1818 (55.2)	0.871(0.782–0.971)	0.521
≥ 60 yr	698 (57.3)	1476 (44.6)	0.821(0.745–0.905)	
**Sex**				
Men	750 (61.5)	2175 (66.0)	0.819(0.784–0.897)	0.003
Women	469 (38.5)	1119 (34.0)	0.883(0.783–0.996)	
**Smoking**				
Current	280 (23.0)	829 (25.2)	0.802(0.692–0.928)	0.478
Ex-smoker	185 (15.2)	558 (16.9)	0.875(0.771–0.993)	
Never smoker	754 (61.9)	1907 (57.9)	0.818(0.732–0.915)	
**Alcohol consumption**				
Never	668 (54.8)	1690 (51.3)	0.901(0.817–0.993)	0.003
1–2/week	334 (27.4)	964 (29.3)	0.778(0.677–0.894)	
3–4/week	115 (9.4)	377 (11.5)	0.852(0.674–1.076)	
≥ 5/week	102 (8.4)	263 (8.0)	0.663(0.512–0.858)	
**Diabetes mellitus**				
Yes	1004 (82.4)	2566 (77.9)	0.825(0.761–0.895)	0.390
No	215 (17.6)	728 (22.1)	0.872(0.741–1.026)	
**BMI**				
< 18.5 kg/m^2	30 (2.5)	35 (1.1)	0.578(0.325–1.030)	0.149
18.5–25 kg/m^2	716 (58.7)	1728 (52.5)	0.861(0.783–0.946)	
≥ 25 kg/m^2	473 (38.8)	1531 (46.5)	0.812(0.724–0.912)	
**SBP**				
< 120 mm Hg	153 (12.6)	325 (9.9)	0.925(0.753–1.138)	0.669
120–130 mm Hg	496 (40.7)	1272 (38.6)	0.845(0.754–0.947)	
130–140 mm Hg	395 (32.4)	1087 (33.0)	0.798(0.702–0.908)	
≥ 140 mmHg	175 (14.4)	610 (18.5)	0.822(0.681–0.993)	

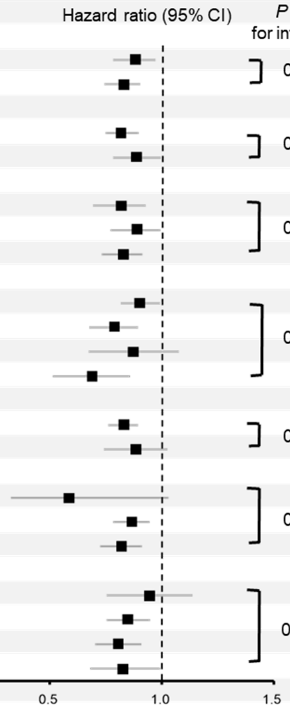

Figure S1. Subgroup analysis. *Fully-adjusted for age; sex, BMI; SBP; the alcohol consumption frequency; the income status; comorbidities, including diabetes, heart failure, and chronic obstructive pulmonary disease; and each antihypertensive mediation use. Square boxes indicate the hazard ratio and horizontal bars indicate 95% confidential intervals. BMI: body mass index; SBP: systolic blood pressure; ARB: angiotensin receptor blocker.

Table S1. International Classification of Disease (10th edition) Clinical Modification (ICD 10-CM) codes used to define the study population, outcomes, and comorbidities.

Diseases	ICD-10 Codes		Diagnosis Definition
Essential hypertension	I10–I13	Essential hypertension<br>I10: essential (primary) hypertension<br>I11: hypertensive heart disease<br>I12: hypertensive chronic kidney disease<br>I13: hypertensive heart and chronic kidney disease	Hospitalization ≥ 1 or outpatient visit ≥ 2
Overall cancer	C00–C96	Site-specific cancer<br>C34: lung cancer<br>C18-C20: colorectal cancer<br>C50: breast cancer<br>C61: prostate cancer<br>C67: bladder cancer<br>C25: pancreatic cancer<br>C64: kidney cancer<br>C22: hepatic cancer<br>C16: gastric cancer	Primary diagnosis and hospitalization ≥ 1
Diabetes	E10–E13	E10: type 1 diabetes mellitus<br>E11: type 2 diabetes mellitus<br>E12: malnutrition-related diabetes mellitus<br>E13: other specified diabetes mellitus	Hospitalization or outpatient visit ≥ 1
Heart failure	I509	I509: heart failure, unspecified	Hospitalization or outpatient visit ≥ 1
Chronic obstructive lung disease	J449	J449: chronic obstructive pulmonary disease, unspecified	Hospitalization or outpatient visit ≥ 1

Table S2. Comparison of baseline characteristics of individuals with and without an incident of cancer during the follow-up period.

Variable	All Patients (N = 70,549)	Without Cancer (N = 66,036)	With Cancer (N = 4513)	*p* value
Follow-up, years	8.6 (6.7–10.8)	8.8 (6.9–10.9)	4.8 (2.6–7.3)	<0.0001
Death, N (%)	3025 (4.3)	1796 (2.7)	1229 (27.2)	<0.0001
Age, years	55.2 ± 9.2	55.0 ± 9.1	58.9 ± 9.0	0.3819
Male sex, N (%)	42,990 (60.9)	40,065 (60.7)	2925 (64.8)	<0.0001
BMI, kg/m2	24.9 ± 3.0	24.9 ± 3.0	24.7 ± 3.0	0.3575
SBP, mmHg	139.7 ± 18.3	139.7 ± 18.3	140.1 ± 19.0	0.0001
Smoking, N (%)				0.0235
Current	16,214 (23.0)	15,105 (22.9)	1109 (24.6)	
Past smoker	12,155 (17.2)	11,412 (17.3)	743 (16.5)	
Never-smoker	42,180 (59.8)	39,519 (59.8)	2661 (59.0)	
Alcohol, N (%)				<0.0001
None	35,575 (50.4)	33,217 (50.3)	2358 (52.3)	
1–2/week	22,265 (31.6)	20,967 (31.8)	1298 (28.8)	
3–4/week	8302 (11.8)	7810 (11.8)	492 (10.9)	
≥5/week	4407 (6.3)	4042 (6.1)	365 (8.1)	
Income, N (%)				0.742
Low (1–3 decile)	17,221 (24.4)	16,113 (24.4)	1108 (24.6)	
Middle (4–7 decile)	23,982 (34.0)	22,430 (34.0)	1552 (34.4)	
High (7–10 decile)	29,346 (41.6)	27,493 (41.6)	1853 (41.1)	
Comorbidities				
Diabetes, N (%)	13,382 (19.0)	12,439 (18.8)	943 (20.9)	0.0006
Heart failure, N (%)	216 (0.3)	199 (0.3)	17 (0.4)	0.3755
COPD, N (%)	1704 (2.4)	1539 (2.3)	165 (3.7)	<0.0001
Use of antihypertensive drugs				
ACEI, N (%)	4210 (6.0)	3848 (5.8)	362 (8.0)	<0.0001
ARB, N (%)	55,645 (78.9)	52,351 (79.3)	3294 (73.0)	<0.0001
BB, N (%)	13,158 (18.7)	12,176 (18.4)	982 (21.8)	<0.0001
CCB, N (%)	51,036 (72.3)	47,593 (72.1)	3443 (76.3)	<0.0001
Diuretics, N (%)	32,990 (46.8)	30,835 (46.7)	2155 (47.8)	0.1687

BMI, body mass index; SBP, systolic blood pressure; COPD, chronic obstructive pulmonary disease; ACEI, angiotensin converting enzyme inhibitor; ARB, angiotensin receptor blocker; BB, beta blocker; CCB, calcium channel blocker.
